# News and Coming Events

**Published:** 1907-07-20

**Authors:** 


### NEWS AND COMING EVENTS.

The International Congress on School Hygiene meets in London in August next from the 5th to the 10th inclusive.

A General Court of Governors of the East London Hospital for Children and Dispensary for Women, Shadwell, E., will be held at the hospital on Monday, July 22, at four o'clock in the afternoon, to receive a report on the general state of the institution from the Board of Management, and to transact such other business as may be deemed desirable.

The festival dinner of the Metropolitan Hospital, Kingsland Road, was held on July 10, at the Hotel Metropole. Mr. Lionel de Rothschild occupied the chair, and in proposing the toast of the evening pointed out that the hospital stands in the very heart of a crowded working-class population, and the patients are supplied by Shoreditch, Hoxton, Hackney, and Islington. Last year the hospital had 1,548 in-patients and 41,642 out-door patients. The great need of the hospital is a home for nurses. Donations to the amount of £4,000 were announced.

Death of Professor Grancher.—By the death of Professor Grancher, at the comparatively early age of sixty-four, France has lost a distinguished authority in pure medical science, and particularly on tuberculosis. Honours had come to him freely, for, besides being Professor at the Faculty of Medicine, he was also a member of the Academy of Medicine and President of the Administrative Council of the Pasteur Institute, having collaborated with Pasteur in his researches. It was, however, against the ravages of tuberculosis that his main studies were directed, and the first attempts to inoculate against this disease were due to his initiative. He was of a very modest and retiring disposition, but inevitably came into prominence in connection with the association which he formed for the Protection of Children against Tuberculosis. After the death of Potain he was the worthiest representative of the best school of French medical thought and activity.

The fourteenth annual meeting of the National Society for Employment of Epileptics was held on July 1 at the London offices, Denison House, Westminster. The chair was taken by Mr. E. Montefiore Micholls. From the medical report it appears that during the past year there have been on the average 197 epileptic "Colonists" in residence at the Society's Colony, besides nine convalescent patients. There was entire freedom throughout the year from any epidemic or infectious disease, and the hon. medical staff again testify to the continued usefulness of the Colony, and generally to the benefits which accrue to the health of confirmed epileptics by the care and management bestowed upon them in the institution, and, they add, "It is now universally admitted that a real necessity exists for special institutions in the treatment of epilepsy, and preferably in the form generally adopted in this and other countries—namely, colonies for epileptics." The report of the Executive Committee states that in response to the appeal recently issued under the auspices of the Prince of Wales as President of the Society, besides other contributions, two most generous offers have been received from intending benefactors, one of whom volunteers to provide the entire cost of building an additional Home at the Chalfont Colony for twenty-four women, while the other undertakes to provide the cost—estimated at £3,500—of building and furnishing a Home for the same number of children. To complete the purposes of the appeal, however, funds are still needed for the erection and equipment of two more Homes and for other purposes.

We are requested to state that Dr. C. O. Hawthorne and Dr. Alex. Morison have resigned their positions as members of the Council of the Scottish Diplomates' Association.

The annual "At Home" of the West Ham and East London Hospital, Stratford, E., will be held on Saturday, July 27, next. The Duchess of Marlborough will lay the foundation-stone of the new extension at 3 p.m. precisely.

A garden fête and sale of work in aid of the funds of the Hospital and Home for Incurable Children, Northcourt College Villas Road, Hampstead, N.W., will be held at the hospital on Saturday, July 20, next, from 3 to 8 p.m. The proceeds of the fête will be devoted to the "Children's League Cot" Fund.

Royal Society of Medicine.—The Therapeutical Section met at Oxford University on Saturday, July 6. The visit was arranged for the Therapeutical Society before it dissolved, and much regret was felt at the absence of Dr. French, who was Hon. Secretary of that Society; he was prevented from being present through illness, which he is happily now recovering from. The members were received at the entrance to Queen's College by Sir Thiselton-Dyer, the first President of the old Society. After a glance at the buildings and some of the other Colleges, he conducted the party to the Museum, where Professor Osler was already waiting ready to point out the beauties of the library and show the way to the laboratories. The first visited was that of Professor Gotch, who explained some of his most recent investigations on the action of the heart. He pointed out that his predecessor, Professor Burdon Sanderson, had conducted his experiments on the empty heart, which revealed that the ventricular contraction began at the base and terminated at the apex. However, Professor Gotch has by delicate electrical experiments on the active heart *in situ* shown that the contraction commences at the base, travels to the apex, and terminates at the aortic base; this was graphically displayed, and many interesting diagrams and photographs were shown. Dr. Ramsden in the same laboratory had arranged a number of beautiful experiments, which he described, determining the "Separation of Solids in the Surface-layers, of Solutions and 'Suspensions.'" Proceeding to the anatomical-rooms, Professor Arthur Thomson showed a thorax with complete transposition of the viscera, and a dissection of a calf's heart, displaying to perfection the auriculo-ventricular bundle (Hiss), and demonstrating how important a structure it is, also some enlarged photographs (stereoscopic) of the very early human embryo. In the pathological-rooms there was an exhibition of macroscopic and microscopic specimens, and Dr. Gibson gave a demonstration of Jacquet's polygraph (modified) on a lad. Professor Osler gave a garden party at his home, to which all were invited; he had laid out for inspection some of his choicest, rare, and interesting folios, which everyone thoroughly enjoyed the privilege of examining. The Section dined in the evening at Queen's College. Sir James Sawyer proposed the health of the President, Dr. T. E. Burton Brown, C.I.E.; he referred to the good work done by the Society of which he was the "Father," remarked on the fitness of seeing him President of the new Section, and hoped for greater things in the future. Dr. Burton Brown replied and thanked the officials of Queen's College for their courtesy in inviting the members to that College, and in placing rooms at the disposal of those who desired to remain until Monday. Dr. Cecil Wall, through whose good offices the visit was rendered possible, acted as cicerone, and showed every attention and kindness to the party.

